# Influence of Cigarette Smoking on Rheumatoid Arthritis Risk in the Han Chinese Population

**DOI:** 10.3389/fmed.2017.00076

**Published:** 2017-06-15

**Authors:** Jian Yin, Dongyi He, Lei Jiang, Fang Cheng, Qian Guo, Shaolan Huang, Xiaofeng Zeng, Yi Liu, Matthew A. Brown, Huji Xu

**Affiliations:** ^1^Department of Rheumatology and Immunology, Changzheng Hospital, The Second Military Medical University, Shanghai, China; ^2^Department of Rheumatology and Immunology, Shanghai Guanghua Hospital, Shanghai, China; ^3^Department of Rheumatology and Immunology, Shanghai Tongji Hospital, Shanghai, China; ^4^Department of Rheumatology and Immunology, Peking Union Medical College Hospital, Peking Union Medical College, Chinese Academy of Medical Sciences, Beijing, China; ^5^Department of Rheumatology and Immunology, Huaxi Hospital, Sichuan University, Chengdu, China; ^6^Institute of Health and Biomedical Innovation, Queensland University of Technology, Princess Alexandra Hospital, Woolloongabba, QLD, Australia; ^7^Translational Research Institute, Queensland University of Technology, Princess Alexandra Hospital, Woolloongabba, QLD, Australia

**Keywords:** smoking, rheumatoid arthritis, anti-CCP antibodies, shared epitope, Han Chinese

## Abstract

**Objectives:**

Cigarette smoking has been shown in European populations to be associated with rheumatoid arthritis (RA) susceptibility. This study aims to examine the association of smoking with RA in the Han Chinese population.

**Methods:**

718 Han Chinese RA patients and 404 healthy controls were studied. The associations of cigarette smoking (current, former or ever vs. never smokers, and pack-years of exposure) with RA, anti-cyclic citrullinated peptide antibody (ACPA) positive RA, IgM rheumatoid factor (RF) positive RA, and baseline radiographic erosions (modified van der Heijde–Sharp scores) were assessed. The interaction between smoking and the HLA-DRB1 shared epitope (SE) in RA was also examined.

**Results:**

In this study, 11 (1.53%) cases and 6 (1.49%) controls were former smokers (*p* = 0.95), while 95 (13.23%) cases and 48 (11.88%) controls were current smokers (*p* = 0.52). Trends toward associations between smoking status (ever vs. never) with RA-overall (*p* = 0.15, OR = 1.44), ACPA-positive RA (*p* = 0.24, OR = 1.37), RF-positive RA (*p* = 0.14, OR = 1.46), or the presence of radiographic erosions (*p* = 0.66, OR = 1.28) were observed although individually here were not statistically significant. There was no evidence of statistical interaction between smoking status (ever vs. never) and SE for all RA, ACPA-positive RA, ACPA-negative RA, RF-positive RA, RF-negative RA (*p* = 0.37, 0.50, 0.24, 0.26, and 0.81 respectively), and the 95% CI for the attributable proportion for all interactions included 0.

**Conclusion:**

This is the first study to examine the association of cigarette smoking with RA in the Han Chinese population. This study shows a trend toward an interaction between smoking and SE carriage influencing the risk of RA, though findings were not statistically significant. It is possible that in the presence of universal exposure to heavy air pollution the effect of smoking on RA risk may be obscured.

## Introduction

Rheumatoid arthritis (RA) is a complex immune-mediated disease that is thought to arise due to the interacting effects of environmental and genetic factors. Several studies performed in populations of European ancestry have suggested that smoking is an environmental risk factor increasing the risk of RA (1–3). Carriage of the HLA-DRB1 shared epitope (SE) is the most important genetic risk factor for RA, accounting for approximately 40% of genetic susceptibility to the disease (4). Several studies have reported that smoking interacts with HLA-DRB1 in increasing the risk of anti-cyclic citrullinated peptide antibody (ACPA)-positive, but not ACPA-negative, RA (5–7). This suggests that the pathogenesis of ACPA-positive RA might differ from that of ACPA-negative disease (8), although the extensive sharing of genetic associations between the two subtypes of RA (9) indicates that they have marked similarity of genetic architecture.

The studies showing that cigarette smoking was associated with RA and ACPA risk have almost exclusively involved populations of Caucasian ancestry. Evidence of association of smoking specifically with ACPA-positive RA has thus far been restricted to studies of European-descent ethnic groups. Studies in Koreans and in African-Americans have found that while smoking increased the risk of developing RA-overall, the association was not restricted to ACPA-positive disease (10, 11). Smoking rates in Chinese are very high, with 52.7% of males being ever smokers. While smoking rates in women are lower (about 2.4%) they are increasing (12). Therefore, it is of great relevance to investigate the role of smoking in the Han Chinese population with RA.

## Materials and Methods

### Patients and Controls

Among these 718 cases, 191 were recruited from Shanghai Changzheng Hospital, 349 from Shanghai Guanghua Hospital, 49 from Shanghai Tongji Hospital, 28 from Peking Union Medical College Hospital, and 91 Huaxi Hospital. All patients were diagnosed in accordance with the American College of Rheumatology 1987 classification criteria (13). 404 healthy controls (blood donors on no prescription medications) were recruited from Shanghai. All human studies have been approved by the Research Ethical Committee of Second Military Medical University, and all patients and controls gave informed written consent for their participation in the studies. Patient charts were reviewed to collect the patients’ demographic information, disease duration, and smoking history before the disease onset. Questionnaires were designed to collect the controls’ demography and smoking information. We defined who ever smoked cigarette as smokers. We also defined ≥10 pack-years as heavy smoker when assessing smoking cumulative effect. EDTA preserved blood was collected from all cases and controls for DNA extraction, and serum samples from all cases. Radiographs of the hands and wrists were obtained at recruitment and scored by two independent experienced radiologists using the modified van der Heijde–Sharp score in 517 of 718 cases. Patients with available scores were classified as having either erosive disease (Sharp erosion score > 0) or non-erosive disease (Sharp erosion score = 0) (14).

IgG ACPA titers were measured by a commercially available second generation (CCP2) enzyme-linked immunosorbent assay (ELISA, EUROIMMUN, Lubeck, Germany) and were considered to be positive at a cut of value >5 IU/ml in accordance to the instructions of manufacturer. IgM rheumatoid factor (RF) was measured by ELISA (EUROIMMUN, Lubeck, Germany) and considered to be positive when the titers were ≥20 RU/ml.

Sequence-specific primer-polymerase chain reaction method was used to determine carriage of HLA-DRB1*01, HLA-DRB1*04, and HLA-DRB1*10 alleles (15), and HLA-DRB1*0101, *0102, *0401, *0404, *0405, *0408, and *1001 were defined as SE.

### Statistical Analysis

Differences of gender, smoking, and SE carriage between groups were investigated using the chi-squared test, and Student’s *t*-test was used to examine the differences of age between cases and controls. Logistic regression was used to examine the association of smoking (ever smokers vs. never smokers, <10 pack-years and never vs. ≥10 pack-years) with affection status, ACPA carriage, RF-IgM positive disease, and baseline radiographic erosions, adjusting for SE status (positive or negative), age, and gender given the differences in these characteristics between cases and controls.

Logistic regression analysis was used to calculate the association of smoking–SE interaction with all patients, carriage of ACPA or RF, and radiographic erosions (16). The attributable proportion (AP) due to interaction (where an AP = 0 corresponds to no interaction and an AP = 1.0 corresponds to “complete” interaction) and corresponding 95% confidence intervals were calculated (17). A *p*-value of <0.05 was considered statistically significant. All analyses were conducted using SPSS13.0 (Chicago, IL, USA).

## Results

Characteristics of cases and controls are shown in Table 1. Cases and controls were well matched for gender (77.44 vs. 75.74% female, *p* = 0.52), but RA cases were older than controls (53.19 vs. 44.56 years, *p* < 0.01). The mean disease duration from onset of symptoms in cases was 7.54 (8.14) years. HLA-DRB1 SE positivity was much higher in RA cases than in controls (40.11 vs. 19.06%, odds ratio 15.8, *p* = 4.3 × 10^−84^). The differences of SE subtypes between two groups were shown in Table 2. 566 (78.83%) and 574 (79.94%) cases were ACPA and RF-positive, respectively. 462 cases (89.36%) had baseline radiographic erosions. 11 (1.53%) cases and 6 (1.49%) controls were former smokers while 95 (13.23%) cases and 48 (11.88%) controls were current smokers. Smoking status was not associated with affection status (*p* = 0.95 and *p* = 0.52 for former and current smokers, respectively), even among heavy smokers (defined as ≥10 pack-years; 13.79 vs. 11.39%, *p* = 0.25).

**Table 1:** Characteristics of Chinese Han rheumatoid arthritis (RA) cases and healthy controls; means (±SD) or %.

	RA cases (*n* = 718)	Controls (*n* = 404)	*p*-Value
Female sex	556 (77.44%)	306 (75.74%)	0.52
Age, years	53.19 (13.61)	44.56 (9.27)	<0.01
Disease duration at baseline visit, years	7.54 (8.14)		
Shared epitope positive	288 (40.11%)	77 (19.06%)	<0.01
Anti-CCP antibody positive	566 (78.83%)	7 (1.70%)	<0.01
RF-positive	574 (79.94%)	81 (20.00%)	<0.01
Bone erosion	462 (89.36%)		
Smoking status			
Never	612 (85.25%)	350 (86.63%)	
Former	11 (1.53%)	6 (1.49%)	0.95
Current	95 (13.23%)	48 (11.88%)	0.52
Cumulative smoking exposure			
Never or <10 pack-years	619 (86.21%)	358 (88.61%)	
Ever ≥10 pack-years	99 (13.79%)	46 (11.39%)	0.25

*CCP, cyclic citrullinated peptide; RF, rheumatoid factor*.

*p-Values calculated using chi-square test or Student’s t-test*.

*201 cases missed the radiographic information*.

**Table 2:** Differences of shared epitope (SE) alleles between rheumatoid arthritis (RA) and healthy control.

SE alleles	RA (%)	Control (%)	*p*-Value	OR (95% CI)
HLA-DRB1*0401	22 (3.06)	18 (4.46)	0.23	0.68 (0.36–1.28)
HLA-DRB1*0404	27 (3.76)	13 (3.22)	0.64	1.18 (0.56–2.30)
HLA-DRB1*0405	165 (22.98)	44 (10.89)	<0.01	2.54 (1.78–3.63)
HLA-DRB1*0408	21 (2.92)	3 (0.74)	0.02	4.03 (1.19–13.59)
HLA-DRB1*0101	19 (2.65)	3 (0.74)	0.03	3.63 (1.07–12.35)
HLA-DRB1*0102	0	0	0	0
HLA-DRB1*1001	64 (8.91)	10 (2.48)	<0.01	3.86 (1.96–7.60)

*Differences of SE alleles were calculated by chi-square test*.

In sex–age–SE adjusted analysis (Table 3), there was no association between smoking status (Table 3, A, former and current vs. never) with either RA-overall (*p* = 0.89 and *p* = 0.13, respectively), ACPA-positive RA (*p* = 0.93 and *p* = 0.20, respectively), RF-positive RA (*p* = 0.87 and *p* = 0.12, respectively), or the presence of radiographic erosions (*p* = 0.81 and *p* = 0.68, respectively). There was also no association between smoking status (Table 3, B, ever vs. never) with RA-overall (*p* = 0.15), ACPA-positive RA (*p* = 0.24), RF-positive RA (*p* = 0.14), or the presence of radiographic erosions (*p* = 0.66). Cumulative smoking (Table 3, C, cumulative exposure vs. never) was also not associated with RA-overall (*p* = 0.15), ACPA-positive RA (*p* = 0.25), RF-positive RA (*p* = 0.12), or radiographic erosions (*p* = 0.55).

**Table 3:** Age–gender–SE adjusted associations of cigarette smoking (smoking status and cumulative smoking exposure) with rheumatoid arthritis (RA) and baseline radiographic erosions in Chinese Han population.

(A) Age–gender–SE adjusted associations of cigarette smoking with RA and baseline radiographic erosions in Chinese Han population							

Smoking status		All RA	Anti-CCP positive RA	Anti-CCP negative RA	RF-positive RA	RF-negative RA	Radiographic erosions
Never		Referent	Referent	Referent	Referent	Referent	Referent
Former	OR (95% CI)	1.09 (0.33–3.54)	0.94 (0.26–3.45)	1.86 (0.34–10.14)	1.11 (0.32–3.85)	1.20 (0.18–7.99)	0.76 (0.08–7.25)
	*p*-Value	0.89	0.93	0.47	0.87	0.85	0.81
Current	OR (95% CI)	1.49 (0.89–2.49)	1.43 (0.83–2.44)	1.37 (0.58–3.23)	1.53 (0.89–2.60)	0.97 (0.42–2.27)	1.29 (0.39–4.23)
	*p*-Value	0.13	0.20	0.47	0.12	0.95	0.68

**(B) Age-gender-SE adjusted associations of ever smoking with RA and baseline radiographic erosions in Chinese Han population**							

**Smoking status**		**All RA**	**Anti-CCP positive RA**	**Anti-CCP negative RA**	**RF-positive RA**	**RF-negative RA**	**Radiographic erosions**

Never		Referent	Referent	Referent	Referent	Referent	Referent
Ever	OR (95% CI)	1.44 (0.88–2.37)	1.37 (0.81–2.31)	1.42 (0.63–3.24)	1.46 (0.88–2.41)	0.99 (0.44–2.25)	1.28 (0.42–3.92)
	*p*-Value	0.15	0.24	0.40	0.14	0.98	0.66

**(C) Age-gender-SE adjusted associations of cumulative smoking with RA and baseline radiographic erosions in Chinese Han population**							

**Cumulative exposure**		**All RA**	**Anti-CCP positive RA**	**Anti-CCP negative RA**	**RF-positive RA**	**RF-negative RA**	**Radiographic erosions**

Never/<10 pack-years		Referent	Referent	Referent	Referent	Referent	Referent
Ever, ≥10 pack-years	OR (95% CI)	1.45 (0.88–2.40)	1.37 (0.80–2.32)	1.47 (0.64–3.38)	1.53 (0.90–2.58)	0.89 (0.38–2.08)	1.31 (0.53–3.24)
	*p*-Value	0.15	0.25	0.36	0.12	0.79	0.55

*CCP, cyclic citrullinated peptide; RF, rheumatoid factor*.

*201 cases missed the radiographic information*.

The assessment of interaction between HLA-DRB1 SE (positive or negative) and smoking (ever or never) in RA was shown in Table 4. After adjustment for age and gender, and considering never smoking/SE-negative as the reference, ORs (95% CI) of ever smoking/SE-negative, never smoking/SE-positive, and ever smoking/SE-positive in all RA were 1.52 (0.88–2.60), 2.82 (2.01–3.95), and 3.46 (1.50–7.95), respectively (*p* = 0.13, <0.01, and <0.01, respectively). In ACPA-positive RA, for the same categories, the ORs (95% CI) were 1.41 (0.79–2.49), 2.86 (2.02–4.06), and 3.62 (1.53–8.55), respectively (*p* = 0.24, <0.01, and <0.01, respectively). In ACPA-negative RA, again for the same categories, ORs (95% CI) RA were 1.70 (0.69–4.19), 3.11 (1.83–5.29), and 2.07 (0.66–10.37), respectively (*p* = 0.25, <0.01, and 0.17, respectively). In RF-positive RA, for the same categories, ORs (95% CI) in RF-positive RA were 1.66 (0.91–2.82), 2.92 (2.07–4.14), and 3.25 (1.36–7.75), respectively (*p* = 0.10, <0.01, and 0.008, respectively), and in RF-negative RA were 0.86 (0.32–2.27), 3.10 (1.81–5.30), and 3.97 (1.88–12.88), respectively (*p* = 0.25, <0.01, and 0.17, respectively). There was no evidence of statistical interaction between smoking status (never, ever) and SE for all RA, ACPA-positive RA, ACPA-negative RA, RF-positive RA, RF-negative RA (*p* = 0.37, 0.50, 0.24, 0.26, and 0.81 respectively), and the 95% CI for the AP for all interactions included 0.

**Table 4:** Assessment of interaction between HLA-DRB1 shared epitope (SE) (positive or negative) and smoking (ever or never) in rheumatoid arthritis (RA) risk in Chinese Han population.

	Never smoking/SE-negative	Ever smoking/SE-negative	Never smoking/SE-positive	Ever smoking/SE-positive	Attributable proportion due to interaction	*p*-Value for interaction

	OR (95% CI)					
All RA	Referent	1.52 (0.88–2.60)	2.82^a^ (2.01–3.95)	3.46^a^ (1.50–7.95)	0.04 (−0.77 to 0.84)	0.37
Anti-CCP antibody positive RA	Referent	1.41 (0.79–2.49)	2.86^a^ (2.02–4.06)	3.62^a^ (1.53–8.55)	0.10 (−0.68 to 0.87)	0.50
Anti-CCP antibody negative RA	Referent	1.70 (0.69–4.19)	3.11^a^ (1.83–5.29)	2.07 (0.66–10.37)	−0.46 (−2.46 to 1.54)	0.24
RF-positive RA	Referent	1.66 (0.91–2.82)	2.92^a^ (2.07–4.14)	3.25^a^ (1.36–7.75)	−0.09 (−1.03 to 0.86)	0.26
RF-negative RA	Referent	0.86 (0.32–2.27)	3.10^a^ (1.81–5.30)	3.97^a^ (1.88–12.88)	0.26 (−0.64 to 1.16)	0.81
Radiographic erosions	Referent	1.22 (0.34–4.38)	1.03 (0.55–1.92)	1.45 (0.28–7.64)	0.13 (−1.45 to 1.72)	0.84

*^a^Statistical differences*.

*Smoking examined as ever vs. never smoking. OR and corresponding 95% CI adjusted for sex, age. AP = 0 corresponds to no interaction and an AP = 1.0 corresponds to “complete” additive interaction*.

*201 cases missed the radiographic information*.

Assessment of interaction between HLA-DRB1 SE (positive or negative) and cumulative smoking exposure (≥10 pack-years, <10 pack-years or never smoker was defined as other) in RA was shown in Table 5. After adjustment for age and gender and considering never smoking/SE-negative as the reference, ORs (95% CI) of heavy smoking/SE-negative, other/SE-positive, heavy smoking/SE-positive in all RA were 1.46 (0.85–2.53), 2.77 (1.99–3.86), and 3.81 (1.53–9.78) (*p* = 0.17, <0.01, and <0.01, respectively). In ACPA-positive RA, these ORs (95% CI) were 1.36 (0.76–2.43), 2.83 (2.01–3.99), and 3.89 (1.50–10.09), respectively (*p* = 0.30, <0.01, and <0.01, respectively) and in ACPA-negative RA were 1.61 (0.65–3.96), 2.98 (1.76–5.05), and 3.24 (0.75–13.92), respectively (*p* = 0.30, <0.01, and 0.12, respectively). Considering RF-positive RA, for the same categories ORs (95% CI) were 1.58 (0.89–2.79), 2.85 (2.02–4.01), and 3.83 (1.47–9.98), respectively (*p* = 0.12, <0.01, and <0.01, respectively), and in RF-negative RA were 0.67 (0.29–2.12), 3.13 (1.85–5.30), and 3.67 (0.99–13.67), respectively (*p* = 0.63, <0.01, and 0.05, respectively). No statistical interaction between cumulative smoking exposure and SE was observed among all RA cases or in ACPA-positive or negative, or RF-positive or negative cases (*p* = 0.61, 0.81, 0.69, 0.62, and 0.75), and the 95% CI for the AP these analyses always included 0.

**Table 5:** Assessment of interaction between HLA-DRB1 shared epitope (SE) (positive or negative) and cumulative smoking exposure (≥10 pack-years, <10 pack-years, or never smoking) in rheumatoid arthritis (RA) risk in Chinese Han population.

	Other^a^/SE-negative	Heavy smoking/SE-negative	Other/SE-positive	Heavy smoking/SE-positive	Attributable proportion due to interaction	*p*-Value for interaction

	OR (95% CI)					
All RA	Referent	1.46 (0.85–2.53)	2.77^b^ (1.99–3.86)	3.81^b^ (1.53–9.78)	0.17 (−0.61 to 0.94)	0.61
Anti-CCP antibody positive RA	Referent	1.36 (0.76–2.43)	2.83^b^ (2.01–3.99)	3.89^b^ (1.50–10.09)	0.18 (−0.61 to 9.69)	0.81
Anti-CCP antibody negative RA	Referent	1.61 (0.65–3.96)	2.98^b^ (1.76–5.05)	3.24 (0.75–13.92)	−0.11 (−1.72 to 1.50)	0.69
RF^b^ positive RA	Referent	1.58 (0.89–2.79)	2.85^b^ (2.02–4.01)	3.83^b^ (1.47–9.98)	0.11 (−0.75 to 0.96)	0.62
RF-negative RA	Referent	0.67 (0.29–2.12)	3.13^b^ (1.85–5.30)	3.67 (0.99–13.67)	0.21 (−0.86 to 1.28)	0.75
Radiographic erosions	Referent	1.01 (0.55–1.85)	1.59 (0.44–5.70)	1.13 (0.22–5.88)	−0.41 (−3.01 to 2.18)	0.75

*^a^Other was defined as <10 pack-years smoking or never smoking*.

*^b^Statistical differences*.

*Smoking examined as ≥10 vs. <10 pack-years or never smoking. OR and corresponding 95% CI adjusted for sex, age. AP = 0 corresponds to no interaction and an AP = 1.0 corresponds to “complete” additive interaction*.

*201 cases missed the radiographic information*.

As shown in Tables 4 and 5, ORs of ever smoking/SE-negative, never smoking/SE-positive, and ever smoking/SE-positive for radiographic erosions were 1.22 (0.34–4.38), 1.03 (0.55–1.92), and 1.45 (0.28–7.64), respectively, and for heavy smoking/SE-negative, other/SE-positive, and heavy smoking/SE-positive were 1.01 (0.55–1.85), 1.59 (0.44–5.70), and 1.13 (0.22–5.88), respectively (all *p* > 0.05). There was no smoking (cumulative smoking)–SE interaction for radiographic erosions (*p* = 0.84 and 0.75, respectively), and the 95% CI for the AP for all these analyses also included 0.

## Discussion

To the best of our knowledge, this is the first study to evaluate the association of cigarette smoking with RA in the Han Chinese population. The results of our study showed that while smoking was not significantly associated with RA, serological positive RA, or radiographic erosions in Chinese patients trends toward associations were noticed in nearly all analyses. Based on previous studies (5–7), Klareskog et al. hypothesized that smoking might trigger citrullination in the lungs of the individuals who carry the HLA-DRB1 SE, thereby providing a substrate for immune activation that finally causes ACPA-positive RA (8). However, epidemiology studies from Korean, Japan, and China have previously not shown any association between smoking, HLA-DRB1, and ACPA-positive RA (9, 18, 19). In this study, we have recruited 718 cases and 404 healthy controls, and logistic regression analysis can remove this bias caused by age and gender (16). In our study, smoking (either ever compared with never smokers, or cumulative dose) was non-significantly associated with both risk of RA-overall, and among ACPA-positive and -negative and RF-positive and RF-negative cases, and presence of baseline erosions. We do not see a formal interaction between smoking and SE carriage in the development of ACPA-positive disease. However, greater increases in risk were seen for SE-positive smokers compared with either SE-negative smokers or SE-positive non-smokers for RA-overall, ACPA-positive and -negative RA, and RF-positive and -negative RA, considering either ever compared with never smokers or cumulative smoking exposure. These findings, while individually not statistically significant, are consistent with and support the presence of interaction between smoking, SE carriage, and the risk of RA. The similarity of findings of the effects of smoking and SE carriage between ACPA-positive and -negative RA suggests that the interaction is not restricted to ACPA-positive disease, as originally suggested.

Based on previous work, there were several significant differences in the role of SE between Han Chinese and Caucasians. First, the prevalence of SE in Han Chinese is much lower than in Caucasians (Han 40% vs. Caucasians 70%) (5–7). Second, the SE subtypes found in Han Chinese and European-descent RA cases were different. European-descent RA cases have two main subtypes, HLA-DRB1*0401 and HLA-DRB1*0405, while Chinese have one main subtype, HLA-DRB1*0405. The prevalence of HLA-DRB1*0401 among Han Chinese and Asians is much lower than that in Caucasians (20). Interethnic *HLA-DRB1* subtype differences like this may explain the differences in our findings compared with those previously reported in European populations.

Another potential explanation for the different results in our study compared with previous studies is the proportion of smokers among RA cases. In studies that involving American or European subjects, more than half of the RA patients and more than 40% healthy people were ever smokers (2, 5). Smoking rates among Chinese women are much lower, with only 2.4% women being ever smokers (12). This is consistent with the low rate of smoking among females in our study. This would lead to lower power to identify an association between smoking and RA and to investigate interactions between smoking, *HLA-DRB1*, and RA.

It is also possible that smoking effects were obscured by high exposure to other environmental agents. For example, personal smoking effects could have been obscured by passive smoking, which we did not assess but which is a major public health problem in China. We also hypothesize that air pollution could play a role. While in Europe and USA air pollution was not associated with RA (21, 22), the very heavy air pollution found in major Chinese cities is a possible contributing factor. Indirect evidence only exists to support this hypothesis. First, a new study (23) found association between airway insults and peptide citrullinations in lungs suggesting that smoking-induced inflammation, rather than smoking itself, contributes to an increase in citrullination in lungs. Second, in the Korea population, whose genetic background and SE constitution are similar with Chinese, but where air pollution is not as marked (24), the effect of smoking on RA risk is observed (9). Third, the prevalence of RA was higher in China than that in South Korea [0.37% (25) vs. 0.27% (26)]. This phenomenon may be due to air pollution exposure being more prevalent than smoking. Fourth, smoking (27) as well as fine atmospheric particulates (PM2.5) (24) were reported to be associated with Oxidative Stress, so we speculate that high concentration of PM2.5 may obscure the effect of smoking. Clarifying the relationship between PM2.5 and RA will help us learn more about the pathogenesis of RA.

In conclusion, this study provides support to the existence of interaction between smoking and *HLA-DRB1* in the pathogenesis of RA. However, the effect is not as strong as reported in other East Asian countries or in European or North American studies. This may be related to statistical power, or an alternative explanation that greater exposure to other potential environmental factors may act as triggers for the disease, such as passive smoking and industrial air pollution, in China. Further studies will be required to investigate the potential role for these factors in RA.

## Ethics Statement

This study was carried out in accordance with the recommendations of the Second Military Medical University Ethics Committee with written informed consent from all subjects. All subjects gave written informed consent in accordance with the Declaration of Helsinki. The protocol was approved by the Second Military Medical University Ethics Committee.

## Author Contributions

HX and MB designed this study; HX organized this study and was the correspondence author; JY and DH carried on this research, they collected samples, demographic data, and finished the lab job, JY also finished the statistic work; LJ, QG, SH, FC, XZ, and YL collected samples and demographic data; HX, MB, and JY wrote the paper. JY and DH contributed equally to this work.

## Conflict of Interest Statement

The authors declare that the research was conducted in the absence of any commercial or financial relationships that could be construed as a potential conflict of interest. The reviewer, HM, and the handling editor declared their shared affiliation, and the handling editor states that the process nevertheless met the standards of a fair and objective review.
